# Synthesis and Characterization of Highly Crystalline Bi-Functional Mn-Doped Zn_2_SiO_4_ Nanostructures by Low-Cost Sol–Gel Process

**DOI:** 10.3390/nano13030538

**Published:** 2023-01-29

**Authors:** Dhiraj Kumar Bharti, Rajni Verma, Sonam Rani, Daksh Agarwal, Sonali Mehra, Amit Kumar Gangwar, Bipin Kumar Gupta, Nidhi Singh, Avanish Kumar Srivastava

**Affiliations:** 1 Nanoscale Research Facility, Indian Institute of Technology Delhi, New Delhi 110016, India; 2 CSIR—Advanced Materials and Processes Research Institute, Bhopal 462026, India; 3 Academy of Scientific and Innovative Research (AcSIR), Ghaziabad 201002, India; 4 CSIR—National Physical Laboratory, New Delhi 110012, India; 5 School of Physics, The University of Melbourne, Parkville, VIC 3010, Australia; 6 Department of Materials Science and Engineering, University of Pennsylvania, Philadelphia, PA 19104, USA; 7 Lam Research Corporation, Fremont, CA 94538, USA; 8 Teerthanker Mahaveer University, Moradabad 244001, India

**Keywords:** Zn_2_SiO_4_, nanoparticles, transmission electron microscope, sol–gel, photoluminescence, lattice strain, nanomagnetism

## Abstract

Herein, we demonstrate a process for the synthesis of a highly crystalline bi-functional manganese (Mn)-doped zinc silicate (Zn_2_SiO_4_) nanostructures using a low-cost sol–gel route followed by solid state reaction method. Structural and morphological characterizations of Mn-doped Zn_2_SiO_4_ with variable doping concentration of 0.03, 0.05, 0.1, 0.2, 0.5, 1.0, and 2.0 wt% were investigated by using X-ray diffraction and high-resolution transmission electron microscopy (HR-TEM) techniques. HR-TEM-assisted elemental mapping of the as-grown sample was conducted to confirm the presence of Mn in Zn_2_SiO_4._ Photoluminescence (PL) spectra indicated that the Mn-doped Zn_2_SiO_4_ nanostructures exhibited strong green emission at 521 nm under 259 nm excitation wavelengths. It was observed that PL intensity increased with the increase of Mn-doping concentration in Zn_2_SiO_4_ nanostructures, with no change in emission peak position. Furthermore, magnetism in doped Zn_2_SiO_4_ nanostructures was probed by static DC magnetization measurement. The observed photoluminescence and magnetic properties in Mn-doped Zn_2_SiO_4_ nanostructures are discussed in terms of structural defect/lattice strain caused by Mn doping and the Jahn–Teller effect. These bi-functional properties of as-synthesized Zn_2_SiO_4_ nanostructures provide a new platform for their potential applications towards magneto-optical and spintronic and devices areas.

## 1. Introduction

Multifunctional nanomaterials play an important role in different fields of applied sciences including semiconductor electronics, solar energy, memory devices, and optoelectronics devices for development of efficient nanosensors and nanosystems [1,2,3,4,5,6]. Therefore, tailoring of these nanomaterials for their desired properties is extremely important, especially for applications in advanced portable devices [7,8,9,10,11,12]. There are several nanomaterials which show tunability with size, shape, and properties with variation of doping concentration [9,13,14,15,16]. Zinc silicate especially (Zn_2_SiO_4_) is a promising candidate for applications in flat panel optical displays, UV detectors, gas sensors, adsorption of toxic ions from water, and thin film electro-luminescence devices applications [17,18,19]. Furthermore, Zn_2_SiO_4_ structures have received significant attention due to their unique wide range of multiple color luminescent properties, wide band gap (5.5 eV), and excellent chemical stability [20]. The willemite Zn_2_SiO_4_ host material is heavily used for applications in the field of phosphors for light emitting devices [21].The Zn_2_SiO_4_ is a natural orthosilicate with a phenacite-like structure in which Zn–O tetrahedral and Si–O tetrahedral share corners to form hollow ‘tubes’ parallel to [0001] planes; and it is a well-known host material for potential applications in the area of phosphors for optoelectronics and blue, green, and red color light emitting devices [1,3,20]. The incorporation of transition metal centers and rare earth ions (luminescence centers) in a Zn_2_SiO_4_ host lattice results in a various interesting luminescent property. Various methods such as vapor phase synthesis, high temperature solid-state reaction, and ball milling have been previously reported for synthesis of the Zn_2_SiO_4_ nanostructures. However, these reported methods require complicated procedure, huge thermal energy, high reaction temperature and time, and the synthesized materials exhibit irregular micro structure morphologies which is unsuitable for applications in devices where precise control over shapes and properties is required.

Recently, several new methods have been reported for the synthesis of Zn_2_SiO_4_ including sol–gel, polymer precursor, spray pyrolysis, and hydrothermal to get different luminescence emissions for various potential application [21,22,23,24]. R. Krsmanovic et al. prepared Sm- and Tb-doped Zn_2_SiO_4_ particle by sol–gel method to get reddish orange and pseudo white emissions [25]. H. Wang et al. and R. Pozas et al. prepared the Zn_2_SiO_4_ with Co dopant using hydrothermal method to obtain the blue pigments [26,27]. K Omri et al. reported the yellow phosphor of Mn-doped Zn_2_SiO_4_ using sol–gel method [28]. G. Gao et al. reported the Eu-doped Zn_2_SiO_4_ nanoparticles prepared by using solid state method to get red color emission [29]. Moreover, K. Omri et al., M. Hafeez et al., and W. Zheng et al. recently reported the diamagnetic and ferromagnetic properties from Mn-doped Zn_2_SiO_4_ nanostructures [30,31,32].

In the present investigations, we synthesized Mn-doped Zn_2_SiO_4_ nanostructures phosphors with various doping concentration using the cost effective and facile sol–gel method. Crystal structure, morphology, and photo luminescence properties of Mn-doped Zn_2_SiO_4_ nanostructures were studied. Effect of doping concentration of Mn in Zn_2_SiO_4_ on its structural, morphological, luminescence, and magnetic properties was also studied. Based on this, a correlation of lattice strain with observed magnetic properties and luminescence behavior is presented.

## 2. Materials and Methods

### 2.1. Materials

Manganese acetate (Mn(CH_3_COO)_2_; 99.999%), zinc chloride (ZnCl_2_; 99.999%), tetraethyl orthosilicate (Si(OC_2_H_5_)_4_; 99.999%), and ethanol (C_2_H_5_OH; 99.999%) were procured from Sigma Aldrich Chemicals Pvt Ltd., Delhi, India. Deionized water (DI) was obtained from a Direct-Q 3UV Millipore water purification system. Copper wires and silver paste were used to make the connection and contact, respectively.

### 2.2. Synthesis of Zn_2_SiO_4_ Nanoparticles

**Figure 1:**
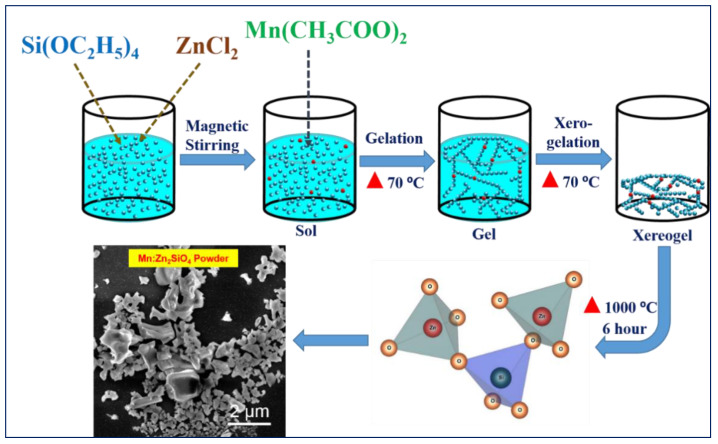
Schematic diagram of sol–gel method for the synthesis of Mn-doped Zn_2_SiO_4_ green phosphor nanoparticles. Red triangle corresponds to heating.

A flow chart for the preparation of Mn-doped Zn_2_SiO_4_ nanoparticles is shown in Figure 1. Mn-doped Zn_2_SiO_4_ nanoparticles were synthesized by sol–gel method under ambient atmosphere followed by thermal annealing. In the first step, 2.0433 mg of ZnCl_2_ aqueous solution was prepared using 16 mL H_2_O and 64 mL EtOH. Then, “x” amount (x = 0.03, 0.05, 0.1, 0.2, 0.5, 1.0, 2.0 wt%) of Mn(CH_3_COO)_2_ was dissolved into main solution. The prepared solution was mixed in a magnetic stirrer at 400 rpm at room temperature for 1 hr. Further, 2 mL of TEOS was slowly added drop by drop using micropipette while stirring after 1 h. Then, the solution was heated at 70 ^0^C resulting, initially in the formation of a white gel and then into a powder when the gel was left overnight. This white powder was then calcined at 1000 °C in box furnace under natural atmospheric pressure for 6 h. Finally, the resultant white Zn_2_SiO_4_ nanoparticles was washed with distilled water and collected for characterization.

### 2.3. Measurement and Characterizations

The structural properties of the prepared samples were measured by X-ray diffraction (XRD) technique using a Rigaku (ultima IV model, Japan) benchtop X-ray diffractometer equipped with a monochromatic Cu-Kα radiation (λ = 1.541 Å) X-ray source. The morphology of the samples was analyzed by high resolution transmission electron microscopy (HR-TEM) JEM 2100F operating at 200 keV and the attached GATAN (Version: GMS 2.32) digital-micrograph software. Elemental mapping was performed using Oxford attachment in HRTEM. The room temperature photoluminescence (PL) investigation was performed using a Perkin Elmer LS-55 fluorescence spectrophotometer with a Xenon (Xe) lamp as the source of excitation. The magnetic properties at room temperature were examined by a SQUID magnetometer (MPMS7 Tesla, Quantum Design, San Diego, CA, USA) in the field range ± 2T with a step size of 400 Oe and a field scan rate of 100 Oe per minute.

## 3. Results and Discussion

### 3.1. Formation of α-Phase Zn_2_SiO_4_

The chemical reaction process and crystal growth mechanism for formation of α-phase Zn_2_SiO_4_ nanostructures can be explained by the following chemical reaction steps:

(1) $$ZnCl_{2}+H_{2}O\;\overset{70\;{}^{\circ}C}{\to }Zn\left(OH\right)Cl+HCl$$

(2) $$Zn\left(OH\right)Cl+C_{2}H_{5}OH\;\overset{70\;{}^{\circ}C}{\to }H_{5}C_{2}O-Zn-OH+Cl_{2}\;↑$$

(3) $$H_{5}C_{2}O-Zn-OH+SiC_{8}H_{20}O_{4}\overset{70\;{}^{\circ}C}{\to }Zn_{4}{\left(OH\right)}_{2}Si_{2}O_{7}.H_{2}O+CO_{2}↑$$

(4) $$Zn_{4}{\left(OH\right)}_{2}Si_{2}O_{7}.H_{2}O\;\overset{550\;{}^{\circ}C}{\to }Zn_{4}{\left(OH\right)}_{2}Si_{2}O_{7}$$

(5) $$Zn_{4}{\left(OH\right)}_{2}Si_{2}O_{7}\overset{750\;{}^{\circ}C}{\to }\beta -phase\;Zn_{2}SiO_{4}$$

(6) $$\beta -phase\;Zn_{2}SiO_{4}\overset{1000\;{}^{\circ}C}{\to }\alpha -phase\;Zn_{2}SiO_{4}$$

The reaction mechanism leading to the formation of zinc silicate can be summarized in the following steps. In the first step, ZnCl_2_ reacts with H_2_O and C_2_H_5_OH at 70 °C and is transformed into Zn (OH)Cl. Then, this Zn(OH)Cl reacts with TEOS, and hydrated hemimorphite leading to formation of Zn_4_(OH)_2_Si_2_O_7_.H_2_O powder. This powder, when annealed at ~550 °C forms dehydrated hemimorphite Zn_4_(OH)_2_Si_2_O_7_. Dehydrated hemimorphite Zn_4_(OH)_2_Si_2_O_7_ is transformed into β-phase ZnSi_2_O_7_ by annealing at ~750 °C and into α-phase Zn_2_SiO_4_ by annealing at 1000 °C.

### 3.2. Structural Analysis

**Figure 2:**
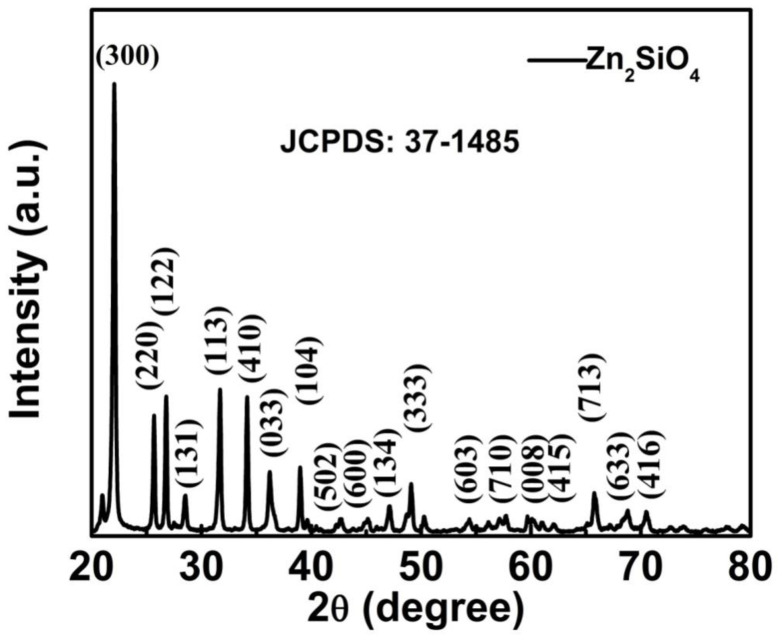
The XRD pattern of Zn_2_SiO_4_ nanostructures annealed at 1000 °C.

The X-ray diffraction (XRD) pattern of the annealed Zn_2_SiO_4_ nanostructures collected at room temperature is shown in Figure 2. All diffraction peaks in the pattern can be indexed to the crystalline willemite (α-Zn_2_SiO_4_, JCPDS card 37-1485) structure of rhombohedral system with lattice parameters a = b = 13.938 Å and c = 9.31 Å, α = β = 90°, γ = 120° revealing the crystallinity of the samples [33].

The average crystallite size of the as grown samples was calculated using the Williamson and Hall method which is a combination of the Scherrer’s equation for size broadening and the Stokes and Wilson expression for strain broadening [34,35,36,37].

(7) $$\beta \cos\theta_{B}=\frac{k\lambda }{D}+4\eta \sin\theta_{B}$$

where β is the full width at half maximum of diffraction peak, η is the strain in the crystallites, and D is the crystallites size [38,39,40]. The constant k is typically close to unity and ranges from 0.8 to 1.39. To calculate the strain and crystallite size, a graph was plotted in between $\beta \cos\theta_{B}\;$and $\sin\theta_{B}$. The strain (η) was then extracted from the slope and the crystallite size was determined from the y-intercept. The average crystallite size of the undoped Zn_2_SiO_4_ nanostructures was found to be 28 nm.

**Figure 3:**
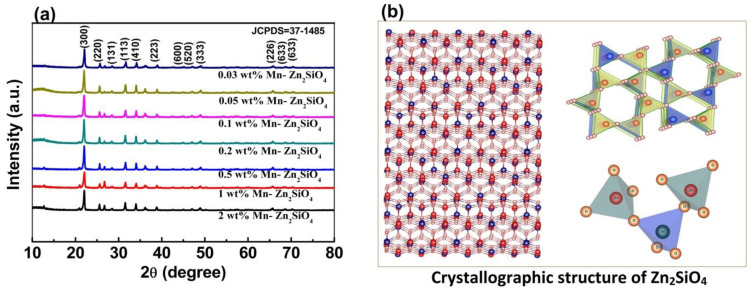
(**a**) XRD patterns of Zn_2_SiO_4_ with different Mn doping concentrations ranging from 0.03 wt% to 2 wt%. (**b**) Unit cell of Zn_2_SiO_4_ and in case of Mn doping, Mn goes to Zn site (Zn^2+^: Red; Si^4+^: Blue; O^2−^:Orange).

The XRD patterns of Zn_2_SiO_4_ samples doped with various doping concentration of Mn are shown in Figure 3a. The result shows that the diffraction peaks of nanostructures shifted towards lower angle with an increase in Mn concentration. This shift in the diffraction peak can be attributed to lattice expansion as a result of induced strain from Mn doping. The likely position of Mn substitution in Zn_2_SiO_4_ lattice is shown in the Figure 3b and it is proposed that Mn^2+^ occupies the Zn^2+^ position. Furthermore, a close view of unit cell can be easily seen from right inset (top and bottom) of Figure 3b.

The different doping concentration of Mn substitution in Zn_2_SiO_4_ lattice and lattice expansion can be easily understood by strain effect as shown in Figure S1. N. Tripathi et al., D.H. Hwang et al., and M.S. Kwon et al. observed the same trend where strain induced effect of Mn in the Zn_2_SiO_4_ nanocrystals enhances the green emission efficiency with short decay time [41,42,43]. The result clearly indicates that the different doping concentration significantly affects the packing of unit cell such as dislocation of host Zn site. Further, the dislocation is confirmed by a Williamson and Hall in the form of strain developed in the lattice which results in slight angle shifts in XRD spectra as shown in Figure 3a. Moreover, depending on different θ positions, the separation of size and strain broadening analysis is done using Williamson and Hall method. Crystal imperfections and distortion of strain-induced peak broadening can be related by the well-known Williamson and Hall method shown in Figure S1.

**Figure 4:**
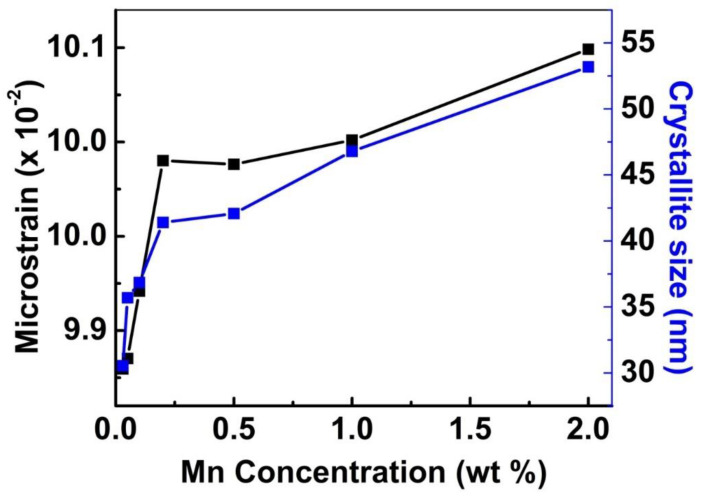
Comparative strain (black square) and crystallite size (blue square) of Zn_2_SiO_4_ as a function of Mn doping in Zn_2_SiO_4_ host lattice.

Figure 4 shows the calculated strain and crystalline size of Zn_2_SiO_4_ as a function of % of Mn doping. It is clearly observed that the strain increases with an increase in Mn doping in the host lattice. The obtained results also indicate that crystallite size increases due to differences in ionic radius between Mn^2+^ and Zn^2+^. (The ionic radii of Zn and Mn are 0.083 and 0.091 nm, respectively).

### 3.3. Morphology Analysis

**Figure 5:**
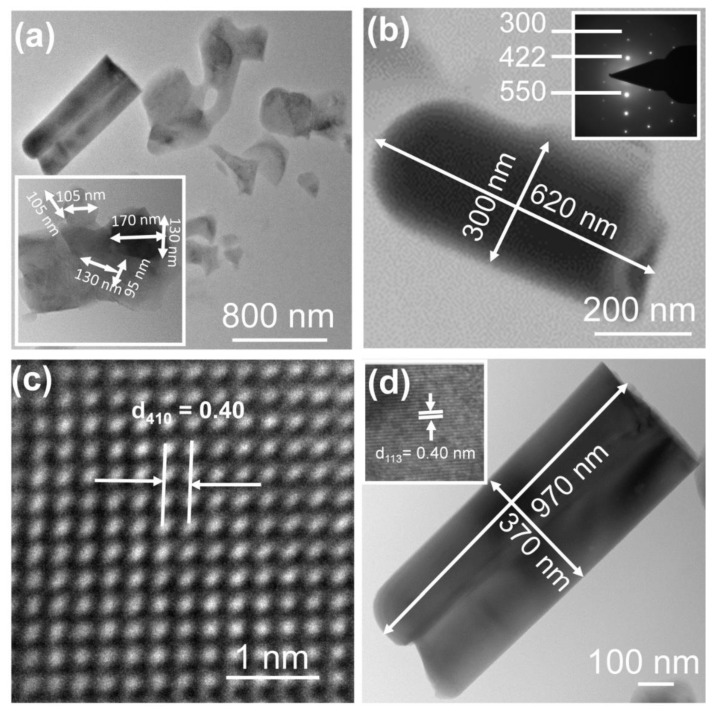
(**a**) Nanorod-like morphology of a 0.2 wt% Mn-doped Zn_2_SiO_4_. (**b**) Magnified view of nanorod-like morphology of individual rod with length 620 nm and width 300 nm. Inset shows the SAED result. (**c**) The HR-TEM image of nanorod. (**d**) HRTEM image of nanoflake-like morphology of individual rod of length 970 nm and width 370 nm. Inset shows the lattice fringe of nanorods corresponding to 113 plane.

The morphology and microstructure of the as-synthesized Zn_2_SiO_4_ nanostructure was analyzed by high-resolution transmission electron microscope (HR-TEM). Figure 5a shows the nanorod-like morphology of 0.2 wt% Mn-doped Zn_2_SiO_4_. The estimation of the dimension of individual nanorods is presented in the inset of Figure 5a. Figure 5b shows the magnified view of nanorod-like morphology of the individual rod with 620 nm length and 300 nm width. Its selected area electron diffraction (SAED) pattern recorded without sample tilting is shown in the inset of Figure 5b. The SAED result reveals that the nanorod is crystalline in nature. The HR-TEM image of the nanorod in Figure 5c shows regular lattice fringes with d-spacing of 0.26 nm, which is in agreement with the inter planar spacing of (300) plane in XRD pattern. Figure 5d shows the magnified view of nanoflake-like morphology of individual rod with length of 970 nm and width of 370 nm. The inset in Figure 5d shows the clear lattice fringe of nanorods which corresponds to 113 plane.

**Figure 6:**
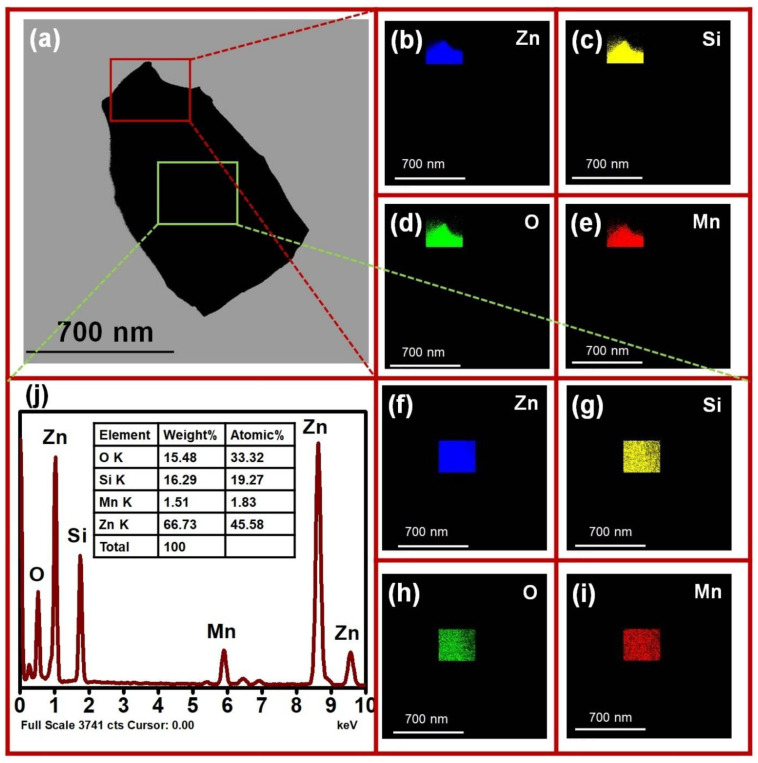
(**b**–**i**) show the spectra obtained from the EDS mapping of (**a**) which confirmed the Mn doping in Zn_2_SiO_4_ lattice. (**j**) shows the quantitative analysis was also carried out to estimate the weight and atomic percentage of samples and EDS spectra.

The elemental mapping of the as-synthesized 0.2 wt% Mn-doped Zn_2_SiO_4_ samples was done by energy dispersive spectroscopy (EDS) added to the HR-TEM instrument. The spectra obtained from the EDS mapping are shown in Figure 6 which confirms the Mn doping in Zn_2_SiO_4_ lattice. The elements present in two different regions of the sample (Figure 6a) are shown in the Figure 6b–e for first region and 6f–i for the second region highlighting the elemental homogeneity of the grown samples. The elemental maps micrographs of Mn, Zn, Si, and O show a uniform distribution over the entire morphology of Mn-doped Zn_2_SiO_4_ nanostructure in both the regions. A quantitative analysis carried out to estimate the weight and atomic percentage of samples is shown in the Figure 6j. The observed spectra show that peak detected at 1.740 eV, 0.525 eV, 8.639 eV corresponds to the Kα_1_ of Si, Kα_1_ of O, and Kα_1_ of Zn, respectively. Further, the peaks detected at 9.572 eV and 1.012 eV correspond to the Kβ_1_ of Zn, and Kα_1_, kβ_2_ of Zn, respectively. The peak observed at 5.899 eV correspond to the Kα_1_ of Mn and peak, 6.491 eV, 0.636 eV corresponds to the Kβ_1_ of Mn and Lα_1_ and Lα_2_ of Mn, respectively [30,44].

### 3.4. Photoluminescence Analysis

**Figure 7:**
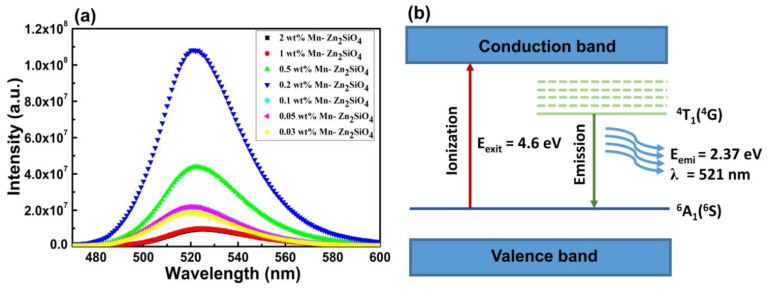
(**a**) shows the broad PL emission band centered at 521 nm of Zn_2_SiO_4_ doped at different concentrations of Mn at an excitation wavelength of 259 nm at room temperature. (**b**) shows a schematic image of shows the green PL emission peak centered at 521 nm transition from the ^4^T_1n_(^4^G) excited state to the ^6^A_1_ (^6^S) ground state.

Figure 7a shows the photoluminescence (PL) spectra of Mn-doped Zn_2_SiO_4_ measured with an excitation wavelength of 259 nm at room temperature (Figure 7b). The emission spectra of the samples show broad green emission centered at 521 nm which arises due to Mn^2+^ ion transitions. The green emission from the sample can be understood in terms of various transition states of Mn element. The green PL emission results transition from the ^4^T_1_(^4^G) excited state to the ^6^A_1_ (^6^S) ground state in 3D orbital electrons of the Mn^2+^ ion [45]. The variation in the PL intensity is due to the size variation and defect level introduced from the doping. It is noted that the nanostructure has large surface area, which significantly impairs the PL intensity by introducing a correspondingly large number of defects. The transition of free electrons in the conduction band by the charge transfer transition of Mn is as described below

(8) $${\mathrm{Mn}}^{2+}\left(3d^{5}\right)\to {\mathrm{Mn}}^{3+}+\left(3d^{4}\right)+{\mathrm{Eg}}_{\mathrm{CB}}$$

These excited free electrons further relax from the conduction band to the ^4^T_1_(^4^G) lowest excitation energy level of the Mn^2+^ ion via non-radiative transitions, followed by a radiative transition from the ^4^T^1^(^4^G) level to the ^6^A_1_(^6^S) ground state or valance state which results in the green PL emission peak centered at 521 nm. A schematic image of the corresponding transition is shown in the Figure 7b. Changing the doping % of Mn^2+^ resulted in only a very small shift in peak position. However, the PL intensity shows nonlinear trend with doping concentration. For better understanding, the PL peak intensity as a function of x at 521 nm was also plotted along with the FWHM as a function of x and is shown in Figure S2. It is clearly observed that the PL intensity first increases with doping concentration with a maxima at 0.2 wt% and then decreases with a further increase in doping. As Mn concentration increases from 0.03 wt % to 0.2 wt%, stress in the crystal lattice also increases as discussed in the XRD section and also shown in Table S1. The decrease of intensity with higher concentration of Mn doping is due to activator concentration quenching and the decrease in the inter-ionic distance between two adjacent Mn^2+^ ion pairs. This reduction of inter-ionic distance between the Mn^2+^ ion pairs leads to exchange of excited free electron migration from one Mn^2+^ ion to another, which causes non-radiative transitions and reduces the emission efficiency and the demonstration at 0.4 wt% Mn doped Zn_2_SiO_4_ is shown in Video V1 [46].

### 3.5. Magnetic Analysis

**Figure 8:**
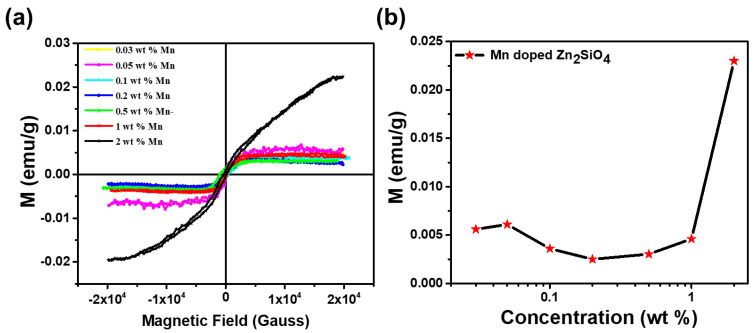
(**a**) M-H plot of Mn-doped Zn_2_SiO_4_ nanoparticles at room temperature. (**b**) Saturation magnetization as a function of Mn wt% in Zn_2_SiO_4_ host lattice.

The M-H plot of Mn-doped Zn_2_SiO_4_ nanorods at room temperature is shown in Figure 8a. It is observed that the value of magnetization increased with increase in the Mn doping concentration, which may be due to the increased amount of impurity in the host lattice of Zn_2_SiO_4_ [47,48]. It was observed in the present study that pristine Zn_2_SiO_4_ exhibits only diamagnetic behavior at room temperature. Further, observed remanence and coercivity for higher Mn content indicates nucleation of ferromagnetic characteristic, whereas diamagnetic behavior was found to be still prevailing for lower Mn^2+^ content. The M-H loops of all the samples were plotted after substituting the diamagnetic contribution of each of the sample [36,37,49]. Magnetization of Zn_2_SiO_4_ with increased Mn doping can be explained in terms of the Jahn–Teller effect which relates the suppressing the spiral spin structure of Zn_2_SiO_4_ by the incorporation of Mn at Zinc sites [30,31]. It is also proposed that Mn cations presented in the Zn_2_SiO_4_ are in mixed valence states of 2+, 3+, and 4+ which could support ferromagnetic coupling through Zener double exchange interaction. In low concentration, the Jahn–Teller effect would be expected to cause a reduction in the tendency toward ferromagnetism since it leads to a splitting of the states. The splitting will reduce the interactions between Mn pairs since hopping requires an extra energy [9,14,34,35,50]. On the other hand, the interaction between Mn–Mn pairs at realistic distances is sufficiently large that it dominates over the Jahn–Teller effect. The interactions between Mn atoms is not greatly affected by lattice relaxations and there is always a clear tendency for ferromagnetic alignment of Mn pairs as evidenced in the present study for higher Mn concentrations. Figure 8b shows value of magnetization with increase in Mn doping wt% concentration into Zn_2_SiO_4_ host lattice.

## 4. Conclusions

We successfully synthesized highly crystalline bi-functional Mn-doped Zn_2_SiO_4_ nanostructures with various doping concentration using a low-cost sol–gel process. The X-ray diffraction results confirmed the rhombohedral phase of Mn-doped Zn_2_SiO_4_ nanostructure. The HR-TEM results confirm the nanorod-like morphology. The PL results show the strong green emission peak centered at 521 nm under 259 nm excitation wavelength. The optimization for highest photoluminescence intensity was achieved by varying different concentrations of Mn-doping in Zn_2_SiO_4_ lattice. We observed that 0.2 wt% Mn doping in Zn_2_SiO_4_ nanostructure showed the highest PL intensity. Magnetic studies confirmed the magnetic nature of Mn-doped Zn_2_SiO_4_ nanostructures and magnetization increased with increase of Mn concentration. Thus, the bi-functional behavior of Mn-doped Zn_2_SiO_4_ nanostructures offers a new avenue to further exploit its potential application in magneto-optical devices.

## Data Availability

The data presented in this study are available in the article.

## Supplementary Materials

The following supporting information can be downloaded at: https://www.mdpi.com/article/10.3390/nano13030538/s1, Figure S1: (a–g) shows the graphs between $\beta \cos\theta_{B}$ and $\sin\theta_{B}$; Figure S2: Comparative strain and crystallite size of Zn2SiO4 as a function of Mn doping in Zn2SiO4 host lattice; Table S1: Effect of Mn doping on the FWHM, strain and crystallites size of Zn_2_SiO_4_ nanostructure; Video S1: The demonstration at 0.4 wt% Mn doped Zn_2_SiO_4_ is shown in Video V1.
