# Supplementary material for: Synthesis and Characterization of Highly Crystalline Bi-Functional Mn-Doped Zn2SiO4 Nanostructures by Low-Cost Sol–Gel Process
Source: Nanomaterials (Basel). 2023 Jan 29;13(3):538. doi: 10.3390/nano13030538 (PMC9921793; doi:10.3390/nano13030538)
Supplement: Supplementary file 1 [file nanomaterials-13-00538-s001.zip › nanomaterials-2044708-supplementary.pdf]

Supplementary Materials

Synthesis and Characterization of Highly Crystalline Bi-Functional Mn-Doped Zn_2SiO_4 Nanostructures by Low-Cost Sol–Gel Process

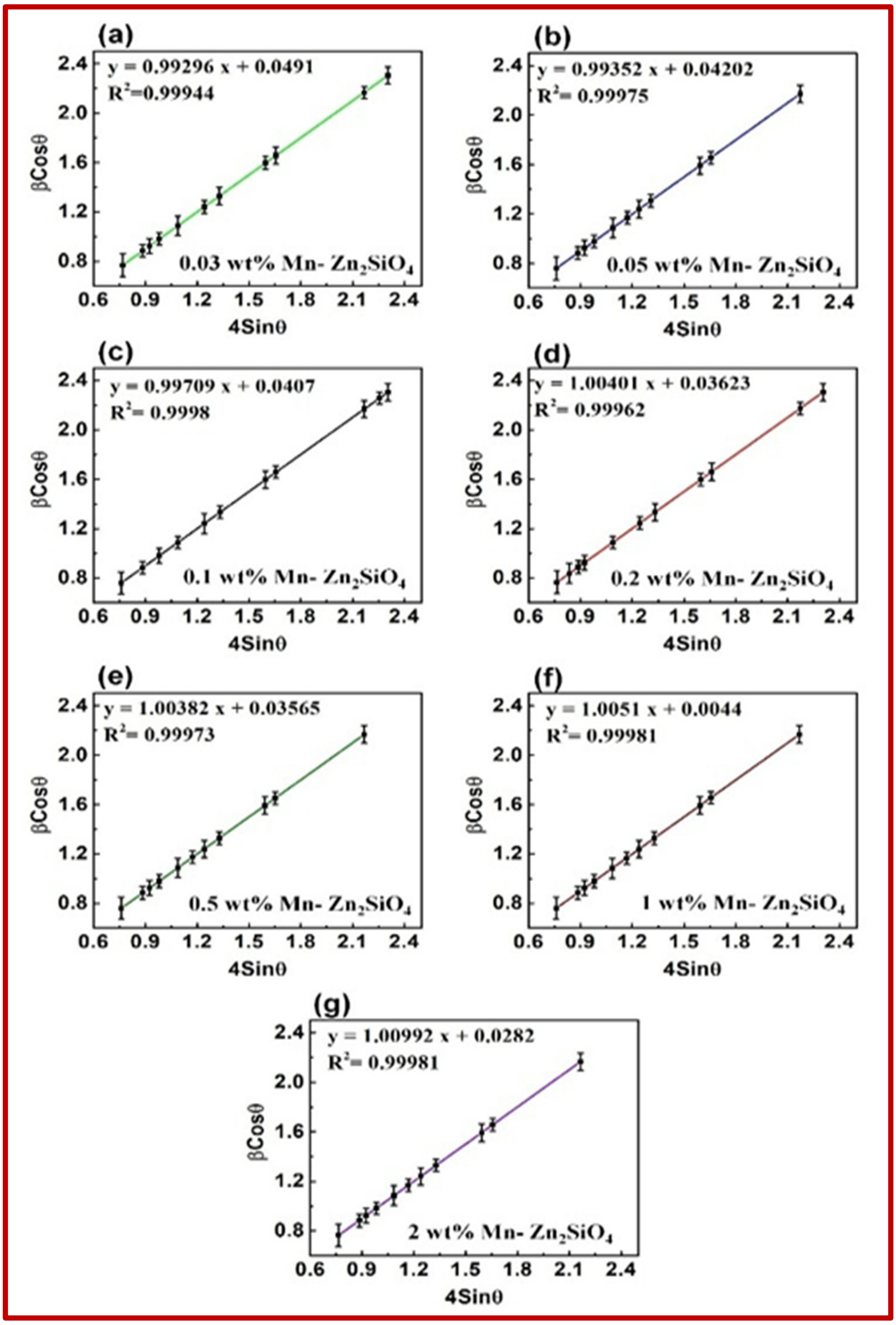

Figure S1(a-g) shows the graphs between $\boldsymbol{\beta \cos \theta_B}$ and $\boldsymbol{\sin \theta_B}$.

Fig. S1 shows the calculated strain and crystalline size of different concentration of Mn doped Zn_2SiO_4. It is clearly observed that strain is increases as Mn doping concentration

increase in the host lattice. The obtained results indicate that crystallite size increases due to ionic radius differences between $Mn^{2+}$and $Zn^{2+.}$ (the ionic radius of Zn and Mn are 0.083 and 0.091 nm, respectively).I am assuming these graphs correspond to different Mn doping concnetrations. This needs to be indicated in the figure description and can also be added as legend in each of the sub-figures.

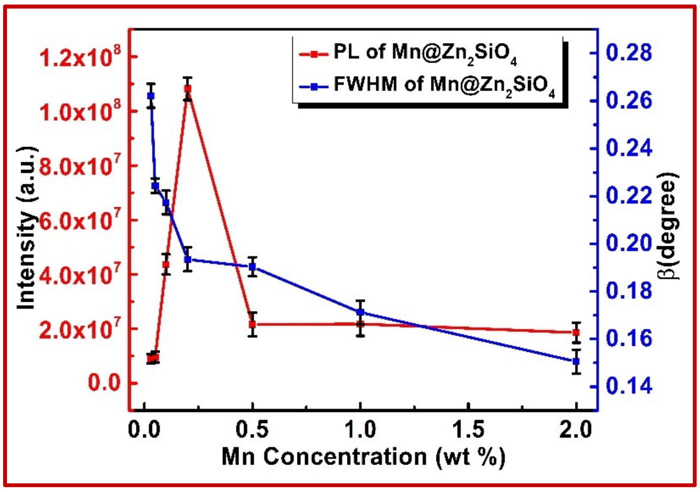

Figure S2. Comparative strain and crystallite size of Zn2SiO4 as a function of Mn doping in Zn2SiO4 host lattice.

Table S1. Effect of Mn doping on the FWHM, strain and crystallites Size of Zn2SiO4 nanostructure

Sr. No.	Mn (wt %)	FWHM β(degree)	Strain	Crystallites Size
**1**	0.03	0.262153801	0.99296	30.5499
**2**	0.05	0.224352455	0.99352	35.69728
**3**	0.1	0.217304673	0.99709	36.85504
**4**	0.2	0.193438563	1.00401	41.40215
**5**	0.5	0.190341808	1.00382	42.07574
**6**	1	0.171174176	1.0051	46.78727
**7**	2	0.15056492	1.00992	53.19149

https://drive.google.com/file/d/1NCbA7lG8Hl9pZz00pj-5YOExEVstGZdh/view?usp=sharing

Video S1: The demonstration at 0.4 wt% Mn doped Zn_2SiO_4
